# Hearing Tests on Mobile Devices: Evaluation of the Reference Sound Level by Means of Biological Calibration

**DOI:** 10.2196/jmir.4987

**Published:** 2016-05-30

**Authors:** Marcin Masalski, Lech Kipiński, Tomasz Grysiński, Tomasz Kręcicki

**Affiliations:** ^1^ Department and Clinic of Otolaryngology, Head and Neck Surgery Wroclaw Medical University Wrocław Poland; ^2^ Department of Biomedical Engineering Wroclaw University of Technology Wrocław Poland; ^3^ Department of Pathophysiology Wroclaw Medical University Wrocław Poland

**Keywords:** hearing test, mobile device, calibration

## Abstract

**Background:**

Hearing tests carried out in home setting by means of mobile devices require previous calibration of the reference sound level. Mobile devices with bundled headphones create a possibility of applying the predefined level for a particular model as an alternative to calibrating each device separately.

**Objective:**

The objective of this study was to determine the reference sound level for sets composed of a mobile device and bundled headphones.

**Methods:**

Reference sound levels for Android-based mobile devices were determined using an open access mobile phone app by means of biological calibration, that is, in relation to the normal-hearing threshold. The examinations were conducted in 2 groups: an uncontrolled and a controlled one. In the uncontrolled group, the fully automated self-measurements were carried out in home conditions by 18- to 35-year-old subjects, without prior hearing problems, recruited online. Calibration was conducted as a preliminary step in preparation for further examination. In the controlled group, audiologist-assisted examinations were performed in a sound booth, on normal-hearing subjects verified through pure-tone audiometry, recruited offline from among the workers and patients of the clinic. In both the groups, the reference sound levels were determined on a subject’s mobile device using the Bekesy audiometry. The reference sound levels were compared between the groups. Intramodel and intermodel analyses were carried out as well.

**Results:**

In the uncontrolled group, 8988 calibrations were conducted on 8620 different devices representing 2040 models. In the controlled group, 158 calibrations (test and retest) were conducted on 79 devices representing 50 models. Result analysis was performed for 10 most frequently used models in both the groups. The difference in reference sound levels between uncontrolled and controlled groups was 1.50 dB (SD 4.42). The mean SD of the reference sound level determined for devices within the same model was 4.03 dB (95% CI 3.93-4.11). Statistically significant differences were found across models.

**Conclusions:**

Reference sound levels determined in the uncontrolled group are comparable to the values obtained in the controlled group. This validates the use of biological calibration in the uncontrolled group for determining the predefined reference sound level for new devices. Moreover, due to a relatively small deviation of the reference sound level for devices of the same model, it is feasible to conduct hearing screening on devices calibrated with the predefined reference sound level.

## Introduction

This paper concerns the calibration of mobile devices for the purposes of the pure-tone audiometry and explores the possibility of the process automation. This study is the first part of the planned research, which aims to define the accuracy of pure-tone audiometry conducted on mobile devices using predefined calibration coefficients.

The evaluation of air conduction hearing threshold may be performed on common electronic devices such as personal computers, laptops, tablets, or mobile phones [1-11]. The examination may not only be applied in screening tests but can also be useful for self-monitoring in the following hearing disorders: fluctuating hearing loss, Menier’s disease, tinnitus, sudden sensorineural hearing loss, age-related hearing loss, or during ototoxic therapy [1,2]. Moreover, it may prove beneficial for preliminary evaluation of patients with otologic complaints [3], by saving the time needed for more sophisticated examinations or for patients who require more medical attention [12]. Mobile phone–based hearing tests can also complement other telehealth methods in otolaryngology such as remote hearing aid adjustment [13] or mobile phone–based otoendoscopy [14]. Hearing examinations conducted on common electronic devices may turn out to be particularly useful in these parts of the world where access to hearing health care professionals is severely limited [15,16].

The air conduction hearing threshold of the pure-tone audiometry can be compared with the self-determined threshold carried out in home setting on common electronic devices in terms of the measurement algorithm, environmental noise, and the calibration method. The accuracy of the automated algorithms for the assessment of the hearing threshold is comparable to that of the ascending method of the pure-tone audiometry [3,5,10,17-19]. Examinations carried out in quiet rooms are characterized by a similar error as those carried out in sound booths [3,4], particularly in patients with hearing impairments [20].

However, the accuracy of hearing examinations conducted in home setting is highly dependent on the calibration method. The device calibration for home-applied self-assessed hearing test may be conducted in various ways, but its omission leads to significant measurement errors [6-9,21]. The device and its components may be calibrated in a laboratory [3-5,10,22,23]. Alternatively, calibration may be conducted in home settings by means of the biological method, that is, in relation to the hearing threshold of a normal-hearing person [1,2,11,24]. The devices calibrated in laboratory conditions exhibit the smallest error, and for those devices, there is no significant statistical difference compared with the pure-tone audiometry [4,5,10,22]. Laboratory calibration may also be conducted for groups of devices that are standardized with similar hardware and software components. In the case of Apple iOS–based devices, the differences in sound intensity between different sets are within 4 dB, making it possible to achieve accurate results in comparison with conventional audiometry [3]. By far, the biggest error is found in the case of biological calibration. The mean standard error of the biological calibration method based on the Bekesy audiometry is estimated at 4.90 dB [24]. However, biological calibration does not require specialized equipment, and its application significantly improves the accessibility of the examination.

Popularization of mobile devices that are offered with bundled headphones entails the possibility of using the once-determined reference sound level in all sets of the same type. Due to the rapidly growing market of mobile devices and their diversity, it seems appropriate to propose a method of automatic determination of the reference sound level without having to calibrate each new device model in a laboratory. The predefined reference sound level determined by means of the biological calibration can meet the aforementioned requirements. Until reliable reference level has been obtained, a user will be asked to perform the biological calibration before the test. This method is particularly important for Android-based devices, which account for about 80.7% of the mobile devices market (data for 2014) [25]. Contrary to iOS-based devices, they do not form a homogeneous group owing to the wide variety of hardware solutions. It can be assumed that tests carried out on the basis of the predefined reference sound level will be more accurate than those based on a single biological calibration. At the same time, the scalability of the proposed solution concerning the number of device models is greater than a laboratory calibration, and therefore, the availability of the test is expected to be higher as well.

In this paper, the reference sound level was compared between the uncontrolled and controlled groups. In the uncontrolled group, the measurements were conducted at home by the users themselves to prepare for further hearing tests. In the controlled group, the audiologist-assisted calibration was performed in a sound booth, in a group of normal-hearing persons verified through the pure-tone audiometry. The test results were compared between the groups to examine the possibility of using the uncontrolled measurements to determine predefined reference sound levels.

## Methods

It was a single-center, parallel trial with no randomization carried out on 2 separate groups of participants: controlled and uncontrolled.

In the uncontrolled group, subjects were recruited via an open access, free mobile phone app “Hearing Test” [26] available on Google Play, designed for conducting hearing examinations on mobile devices. The functionality of the app was presented on the Google Play website so that the participants could learn more before the installation. A prerequisite for using the app was giving consent during the initial launch for the data to be anonymously used for the purposes of app development and scientific work. The main functionality of the app is the assessment of the hearing threshold within the frequency band 250 Hz to 8 kHz in relation to the reference sound level. If the predefined reference sound level for the particular model was not available in the centralized database, the device had to be calibrated before the examination. Calibration could also be conducted to verify the reference sound level used during the examination. Therefore, the calibration measurements, which are subject of interest in this paper, were not the outcome of intended actions but resulted from the wish to conduct the hearing test or to verify the test results. All the users who conducted calibration using the bundled headphones were assigned to the uncontrolled group. Detailed instructions on calibration were presented directly before the measurement. They contained the requirements of conducting the test in silence, on headphones, by a normal-hearing person aged 18 to 35 years. Subjects were identified by means of a unique number assigned to the device, more precisely to the instance of the device’s operational system. When more than 1 calibration was assigned to the same identification number, the results of the last calibration were analyzed. The device model was identified by the manufacturer name and the end-user-visible name for the end product.

In the controlled group, the participants were recruited offline from among the employees and patients of otolaryngology clinic through face-to-face prompting. The eligibility criteria were the possession of an Android-based mobile phone with bundled headphones and willingness to participate in the study. In the first stage, the hearing threshold of the participant was verified through the pure-tone audiometry with the ascending method [27] conducted on a clinical audiometer Interacoustic AD229e with TDH-39 headphones previously calibrated according to the norm ISO 389-1:1998. Having installed the “Hearing Test” app, the calibration was performed in a sound booth with the use of bundled headphones. If the hearing of the participant was within the normal range (ie, no higher than 20 dB HL in the frequency between 125 Hz and 8 kHz), the participant was asked to perform calibration twice under the supervision of an audiologist. If not, calibration was conducted twice by 1 of 2 normal-hearing audiologists. The results of the first calibration were used in the analysis, whereas the other one served for calculating the test–retest difference.

In both the controlled and uncontrolled groups, calibration consisted of determining the hearing threshold by means of the Bekesy audiometry. The Bekesy audiometry is a self-recording hearing test in which the subject controls the intensity of the stimulus by pressing a button, while the frequency of the stimulus is being slowly changed within the audible range. The intensity of a stimulus decreases as long as the button is pressed and increases when released, yielding a near-threshold zigzag tracing. The calibration was carried out using a pulsed tone, within the frequency band 125 Hz to 16 kHz, at frequency change 1 octave/minute and intensity change 2 dB/second [24]. It took 7 minutes to complete the calibration. The results were stored in the centralized database. Calibration coefficients were determined for 250 Hz, 500 Hz, 1 kHz, 2 kHz, 4 kHz, 6 kHz, and 8 kHz by calculating the median within ±1/2 octave of sound intensities, at which a change in the button state occurred. Thus, the calibration coefficients determine the level of the signal generated by the device, which equals the hearing threshold of the reference person.

### Comparison of Reference Sound Levels

The reference sound level has been defined as such a level of the signal generated by the device, which will produce in the bundled headphones the sound at the intensity of 0 dB HL. The reference sound level can be estimated based on calibration coefficients assuming that the hearing threshold of the reference person nears 0 dB HL. The estimation will be much more accurate if the calibration coefficients are decreased by the hearing threshold of the reference person. By the same token, the reference sound level can be estimated on the basis of population if the distribution of its calibration coefficients and the hearing threshold are known.

The groups were compared by means of the reference sound level. In the uncontrolled group, the reference sound level was obtained by decreasing calibration coefficients by the literature-based median of the hearing threshold in the population screened for ear-related disorders and a history of noise exposure [28]. In the controlled group, the reference sound level was calculated as the mean value of calibration coefficients decreased by the pure-tone hearing thresholds. The comparison of reference sound levels in groups was conducted on the basis of confidence intervals (CIs).

The sample size was determined on the basis of the standard deviation (SD) of the difference between reference sound levels, and therefore, it constitutes a set of alternative pairs of numbers determining the required number of calibrations in the controlled and uncontrolled groups. The more calibrations are conducted in one group, the fewer will be required in the other. On the basis of preliminary measurements, for statistical significance .05, statistical power 0.8, and the magnitude of the effect 15 dB, the required number of calibrations for a single model in the uncontrolled group was determined at 60, 25, or 15 on the assumption of 1, 2, or 3 calibrations in the controlled group, respectively. The experiment was completed after satisfying criteria for 10 different models. The number of models was agreed arbitrarily.

### Intramodel and Intermodel Analyses

Additional analyses, that are, intramodel and intermodel analyses, have been carried out. The accuracy of the hearing examination conducted on the basis of predefined reference sound level is dependent on the variability of reference sound level in the group of devices belonging to the same model. On the basis of the results in the controlled group, an attempt was made to estimate the aforementioned variability.

Moreover, the necessity of applying different sound reference levels for different models has been verified. The comparison of the reference sound levels between models was performed based on the data in the uncontrolled group.

The “Hearing Test” app was developed based on Web page–embedded Java applet ported to Android system. The Java applets had previously been used in the research on validity evaluation of self-test Web-based pure-tone audiometry [2] and in the research on biological calibration methods for Web-based hearing tests [24]. During the trial, based on preliminary analyses, the number of calibrations required to calculate predefined calibration coefficients was reduced (from 100 to 16; September 6, 2014). Although the change caused a slower growth of the number of calibrations in the uncontrolled group for models that have exceeded the required threshold, it contributed to improving the app’s ratings in Google Play, increasing the number of users, and thus quickly obtaining the required sample size. The remaining modifications to the app were connected with its development (September 12, 2014) and correction of minor bugs (September 25, 2014). The app’s functionalities were expanded to include the possibility of adding notes, sending and printing the examination results, and adjusting the calibration coefficients on the basis of the pure-tone audiogram. During the trial, the app was made available in 8 new languages besides English and Polish also in German, Spanish, French, Italian, Japanese, Korean, Portuguese, and Russian (September 12, 2014).

## Results

**Figure 1:**
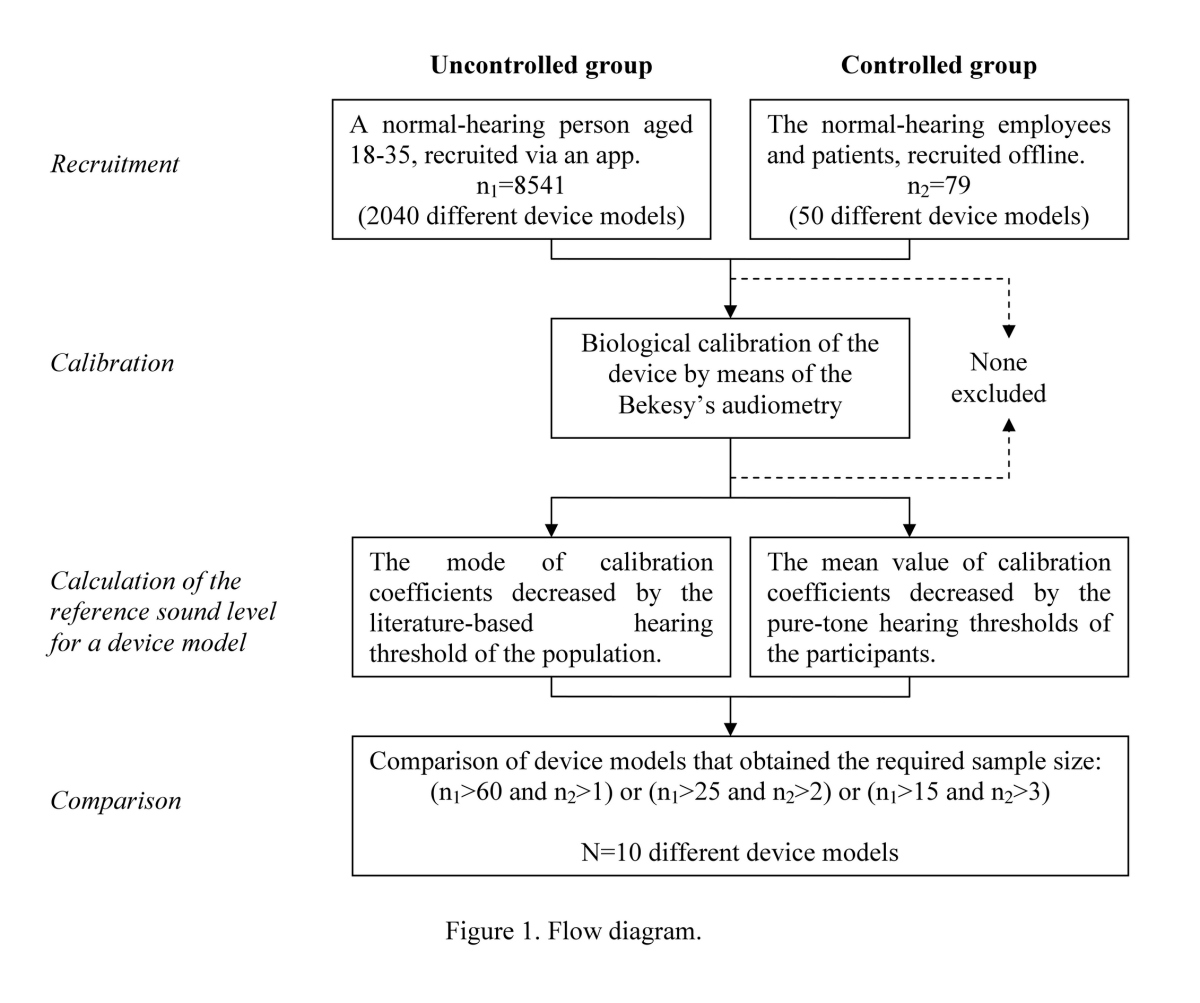
Flow diagram.

In the period between November 30, 2013 and February 13, 2015, the app was installed 114,546 times from Google Play. During that time, 20,747 calibrations were carried out on 18,154 different devices representing 2772 different models. Overall, 8988 of these calibrations carried out on 8620 different devices (2044 different models) were performed on the bundled headphones. In the controlled group, 158 calibrations (test and retest) were conducted on 79 devices representing 50 models. All calibrations conducted on the remaining 8541 devices representing 2040 models, that is, 8830 calibrations, were qualified to the uncontrolled group. In the controlled group, 11 of 79 participants (14%) were found to have slight hearing impairment, and in these cases, the calibration was performed by 1 of the 2 normal-hearing audiologists. The analysis was performed on 10 models for which the required sample size was obtained, that is, at least 60, 25, or 15 calibrations in the uncontrolled group in the case of 1, 2, or 3 calibrations in the controlled group, respectively (Figure 1).

### Comparison of Reference Sound Levels

**Figure 2:**
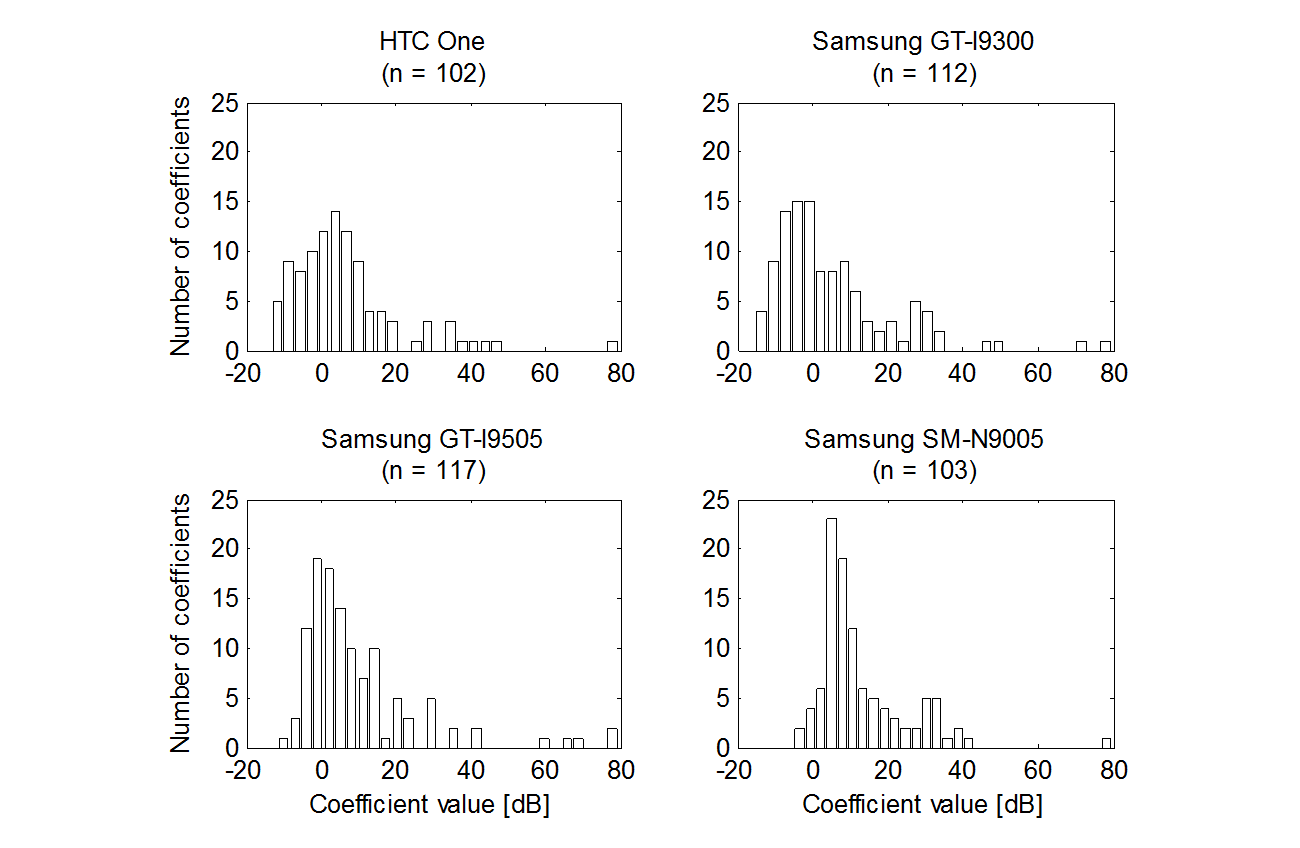
Calibration coefficients for sample device models at 1 kHz in the uncontrolled group.

In the uncontrolled group, the central tendency of calibration coefficients was estimated by the mode as it is more robust than the median to outliers [29-31]. Hence, no data were excluded from further analysis. Choosing the mode as a measure of the central tendency was dictated by significant asymmetry in the distribution (Figure 2), whose causes should be sought in greater probability of error, leading to overestimation of the coefficient rather than its underestimation (see Discussion). The mode was determined by the mean of shortest half sample [29] and its SD by the bootstrap method [32].

In the uncontrolled group, the reference sound level was obtained by decreasing the mode of calibration coefficient by the median of the hearing threshold level for the population meeting the calibration requirements, that is, aged 18 to 35 years with no prior hearing problems. The median was estimated on the literature-based data of the hearing threshold in population screened for ear-related disorders and a history of noise exposure [28]. The median for the group aged 18 to 35 years was calculated as the weighted arithmetic mean under the assumption that the hearing threshold in the group aged 18 to 19 years is similar to that in the group aged 20 to 29 years (Table 1). Due to considerable size of the trial sample (n=5498) used to determine the median value, its error was considered negligible.

In the controlled group, the reference sound levels were determined by decreasing the values of calibration coefficients by the pure-tone hearing thresholds. The SD of the reference sound levels was estimated by means of the differences between devices of the same model. The differences were calculated at all 7 frequencies. The SD of the difference was obtained at the level of 9.09 dB (95% CI 8.67-9.55), and thus, the SD of the reference sound level in the controlled group was estimated at 6.43 dB (95% CI 6.13-6.75).

**Figure 3:**
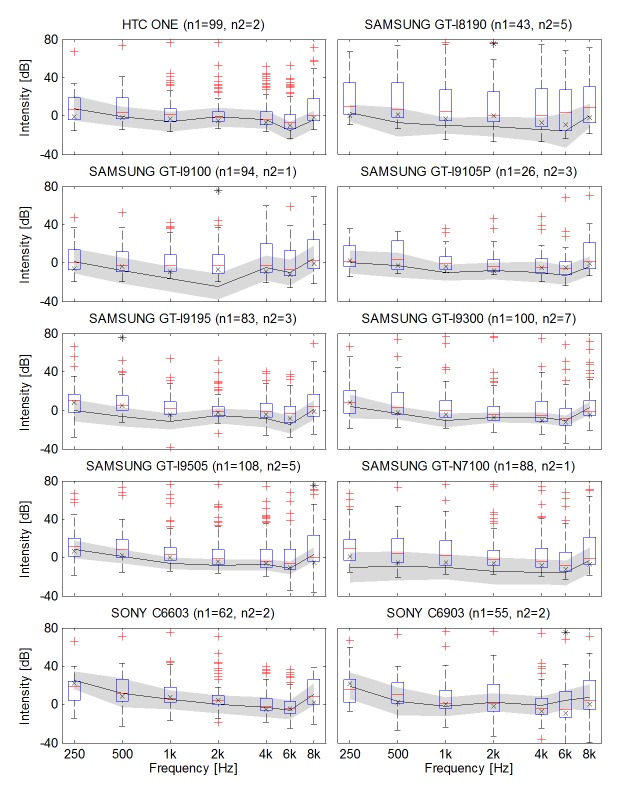
The reference sound level for different device models (boxplot—calibration coefficients in the uncontrolled group decreased by the median of the hearing threshold in the population of normal-hearing subjects, x—the reference sound level in the uncontrolled group, that is, the mode of boxplot data, continuous line—the reference sound level in the controlled group, gray area—95% confidence interval for the difference between reference sound level in groups, *—statistically significant difference).

The reference sound levels in both the groups are presented in Figure 3. They are expressed by the intensity of digital signals, which generate sounds at the level of 0 dB HL. It has been arbitrarily assumed that the lowest possible signal level that can be theoretically generated, whose intensity results directly from quantization, will be at the level of −40 dB. With the aforementioned assumptions, for most mobile devices, the signals with the frequency of 1 kHz and intensity of 0 dB generate sounds close to the level of 0 dB HL.

Reference sound levels in the uncontrolled group were compared with the reference sound levels in the controlled group (Figure 3,Tables 2 and 3). CIs were estimated on the basis of the SD of the difference between these values (Multimedia Appendix 1). At 95% CIs, statistically significant differences were found in 5 of 77 (7%), which is in accordance with the statistical significance level and confirms the similarity of reference sound levels in both the groups. The mean value of the differences calculated jointly for all the frequencies and all the analyzed models was 1.50 dB (SD 4.42).

**Table 1:** Literature-based medians of the hearing threshold by gender and groups aged 20 to 29 years and 30 to 39 years [28] and the estimated median of the hearing threshold for the group aged 18 to 35 years.

			Hearing threshold median (dB HL)						
Gender, age	N	Weight	250 Hz	500 Hz	1 kHz	2 kHz	4 kHz	6 kHz	8 kHz
Men, 20-29	650	12	12.5	5.0	2.5	2.5	5.0	10.0	7.5
Women, 20-29	1840	12	12.5	7.5	2.5	2.5	2.5	10.0	7.5
Men, 30-39	619	5	12.5	7.5	5.0	5.0	10.0	15.0	10.0
Women, 30-39	2389	5	15.0	7.5	5.0	5.0	5.0	12.5	10.0
Estimated median, 18-35.			12.9	6.6	3.2	3.2	4.9	11.1	8.2

**Table 2:** Differences in reference sound levels between uncontrolled and controlled groups by device models.

Model	Number of calibrations in the uncontrolled group	Number of calibrations in the controlled group	Difference between reference sound levels in groups, dB (SD)
HTC ONE	99	2	−0.75 (4.58)
SAMSUNG GT-I8190	43	5	4.99 (5.16)
SAMSUNG GT-I9100	94	1	1.29 (3.92)
SAMSUNG GT-I9105P	26	3	3.35 (3.06)
SAMSUNG GT-I9195	83	3	5.80 (4.25)
SAMSUNG GT-I9300	100	7	−0.04 (4.29)
SAMSUNG GT-I9505	108	5	0.45 (4.43)
SAMSUNG GT-N7100	88	1	4.78 (4.28)	
SONY C6603	62	2	−1.14 (4.31)
SONY C6903	55	2	−3.73 (5.94)

**Table 3:** Differences in reference sound levels between uncontrolled and controlled groups by frequencies (number of models n=10).

Frequency (Hz)	Difference between reference sound levels in groups, dB (SD)	
250	1.58 (5.91)
500	2.63 (4.59)
1k	4.68 (2.86)
2k	2.15 (4.41)
4k	0.53 (4.13)
6k	0.67 (6.48)
8k	−1.74 (5.72)

### Intramodel Analysis

The reference sound level in the controlled group was measured by decreasing the value of calibration coefficient by the pure-tone hearing threshold of the reference person. Therefore, the variability of the measured value of the reference sound level is dependent on the variability of the real value of the reference sound level, the measurement error of the pure-tone hearing threshold, and the measurement error of calibration coefficient. Assuming the literature-based SD of the test–retest examination for pure-tone audiometry at 5.37 dB (95% CI 5.02-5.77) [24], the error of determining pure-tone hearing threshold is 3.80 dB (95% CI 3.55-4.08). The measurement error of the calibration coefficient was determined directly on the basis of the obtained results. The SD in the test–retest examination was found to be 4.62 dB (95% CI 4.37-4.91), and thus, the error is 3.27 dB (95% CI 3.09-3.48). Finally, using the variability of the measured value of the reference sound level described in the previous paragraph, the variability of the real value of the reference sound level was expressed by SD of 4.03 dB (95% CI 3.93-4.11; Multimedia Appendix 2).

### Intermodel Analysis

**Figure 4:**
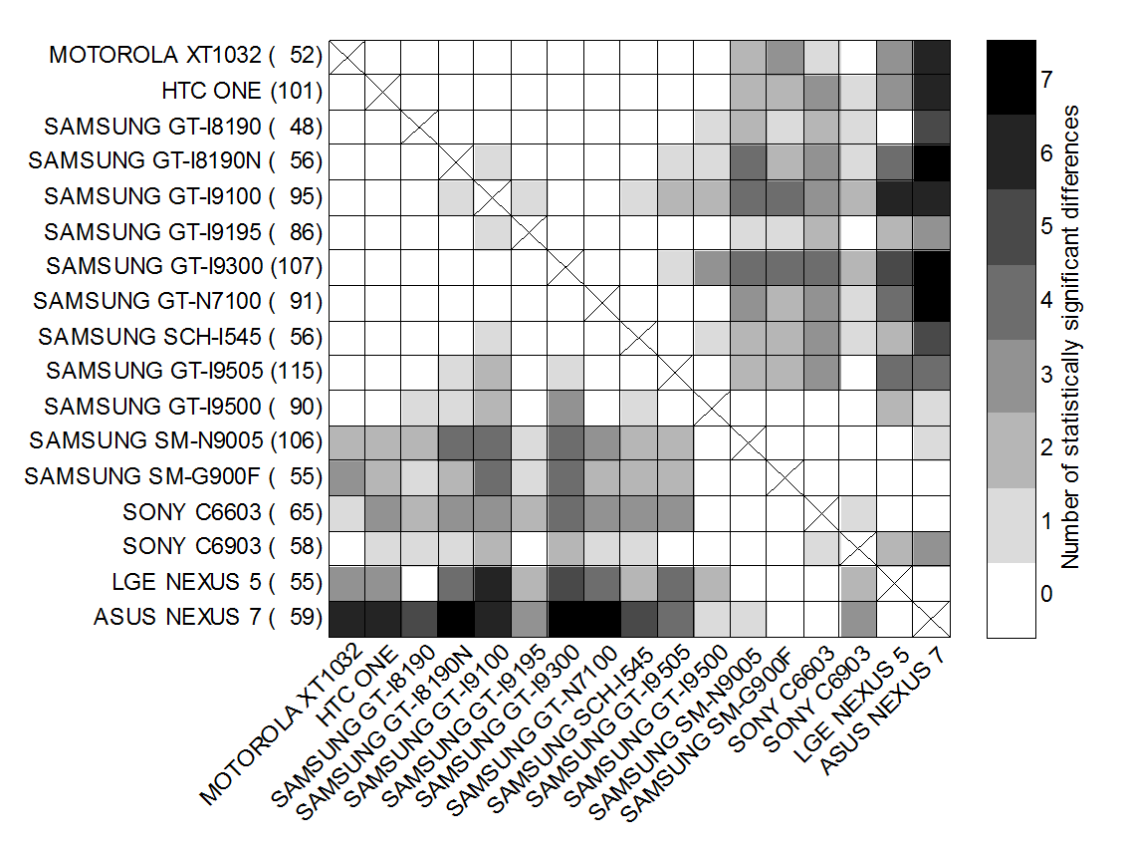
Comparison of reference sound levels between models by means of the number of frequencies with statistically significant differences (*P*=.01).

Reference sound levels were compared between models based on the measurements carried out in the uncontrolled group. The distributions of differences between modes of reference sound levels were determined using the bootstrap method. The comparison was conducted at the level of statistical significance *P*=.01 based on the critical value determined by SD of the bootstrap distribution. Statistically significant differences were found in reference sound levels (Figure 4).

## Discussion

Reference sound levels determined for the purpose of the hearing test were compared between controlled and uncontrolled groups for different models of mobile devices. The difference between reference sound levels was obtained at 1.50 dB (SD 4.42), which confirms the possibility of using calibration in the uncontrolled group for determining predefined reference sound level for new devices.

The mean, real variability of the reference sound level in the group of devices belonging to the same model was expressed by the SD estimated at 4.03 dB (95% CI 3.93-4.11). This value limits the hearing test accuracy when a common, predefined reference sound level is applied to all devices of the same model. The estimated variability of reference sound level is higher than the value determined for iPads, for which the maximum intrafrequency difference was 4 dB [3]. One of the reasons for such a difference could be the change in frequency characteristics of headphones during their usage. In the conducted measurements, new headphones were used as well as long-used ones. The variability determined previously is, however, significantly smaller than the error of the biological method based on the Bekesy audiometry estimated at the level of 4.90 dB (95% CI 4.46-5.45) [24].

Statistically significant differences were found in the reference sound levels between models, in particular between models produced by different manufacturers. At the same time, some models had similar reference sound levels. This confirmed the need to determine reference levels individually for various groups of devices, which does not necessarily correspond with the proposed division that was based on manufacturer of the product and the end-user-visible name for the end product.

Distribution of calibration coefficients in the uncontrolled group was characterized by significant asymmetry caused by higher number of coefficients with potentially overstated values. This may be related to greater odds of making a mistake leading to overstating coefficient values rather than understating them. The overstated value of the coefficient may be caused by potential errors such as noise during calibration, hearing deficits of the reference person, calibration on damaged headphones, or abandoning the device during calibration. The understated coefficient values may only be influenced by interferences or other disruptions when they are misinterpreted as a reference signal. The mean difference of the reference sound level between groups calculated on the basis of the mode was 1.50 dB (SD 4.42). However, if a median is used instead of a mode, the mean difference will be 5.39 dB (SD 3.93), which proves the validity of the applied measure of the central tendency.

**Figure 5:**
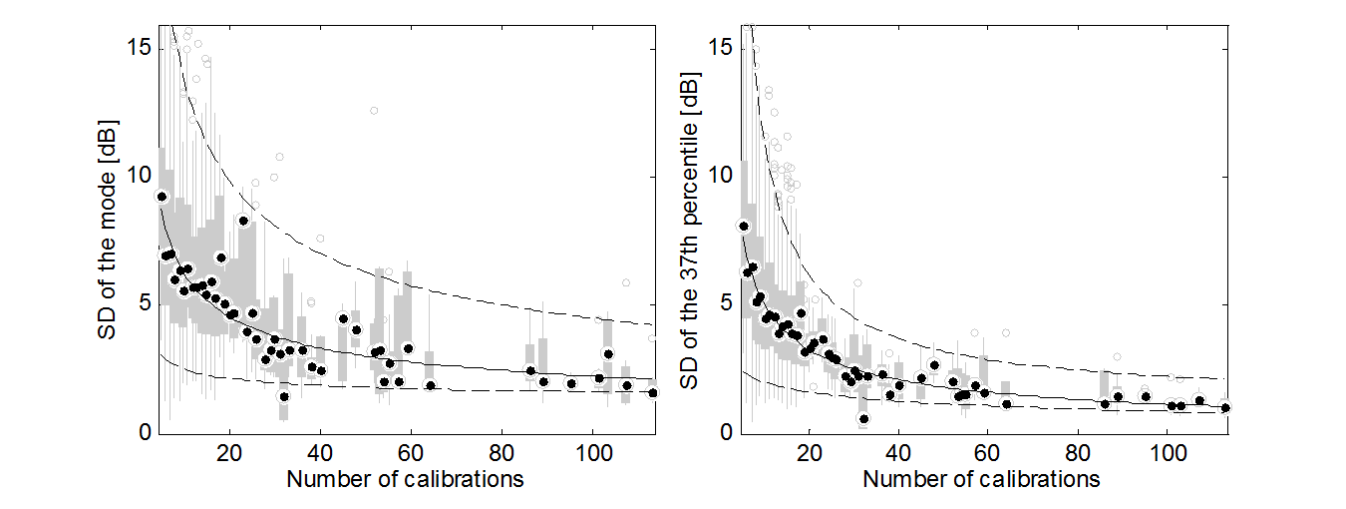
Standard deviation of reference sound level in relation to the number of calibrations (continuous line—median; dotted lines—percentiles: 2.5% and 97.5%).

Predefined reference sound levels for new device models may be determined semiautomatically based on the calibrations conducted in the uncontrolled group. To estimate the error of the determination, the SD of the mode of reference sound level was calculated in relation to the number of calibrations (Figure 5). Calculations were carried out for every model, cumulatively for all frequencies, using the bootstrap method. The mean standard error of determining the predefined reference sound level was found below 5 dB for the number of calibrations greater than 16.

The mode value precisely determines the reference sound level but turns out to be not stable enough with a small number of calibrations. Therefore, an attempt has been made to determine the reference sound level using a quantile, which in most cases included the mode. The modal quantile has been found at the 37th percentile. The mean difference between the groups, determined on the basis of the 37th percentile, is comparable to the value obtained for the mode and equals 1.02 dB (SD 3.99). At the same time, the 37th percentile is characterized by significantly smaller SD (Figure 5). This is particularly important in the case of a small number of calibrations. For the 37th percentile, the mean standard error of determining the predefined reference sound level was found below 5 dB for the number of calibrations greater than 10.

The accuracy of hearing examinations determines their application. Therefore, an attempt was made at estimating the SD of the difference between classical pure-tone audiometry and a hearing test conducted on a mobile device calibrated by means of predefined sound level that was determined in uncontrolled settings using the biological method. Assuming the standard error of determining the predefined reference sound level for 16 calibrations at 5.08 dB (95% CI 2.25-11.02), variability of reference sound level within a single model at 4.03 dB (95% CI 3.93-4.11), the literature-based SD of test–retest differences of the pure-tone audiometry at 5.37 dB (95% CI 5.02-5.77) [24] and making a conservative assumption of independence of the aforementioned variables, we obtain the SD of the difference at 8.42 dB (95% CI 6.76-13.16) (Multimedia Appendix 3). The aforementioned value is comparable to the values of the screening methods, which had been presented in earlier works [1,2] and thereby validates the application of the method in hearing screening.

The method presented in this paper can be applied in screening hearing examinations on a large scale with the use of popular mobile devices sold with bundled headphones. Due to rapidly growing market of mobile devices, the main advantage of the method is the semiautomated calibration of new models. Predefined reference sound level for a new model may be determined on the basis of a biological calibration conducted by the first users of devices. To confirm the estimated accuracy of the method, it is advisable to conduct a direct comparison of pure-tone audiometry and a hearing test on mobile devices calibrated biologically by means of the predefined reference sound level.

## Appendix

Multimedia Appendix 1 Standard Deviation of the Difference Between the Mode of the Reference Sound Level in the Uncontrolled Group and the Mean Reference Sound Level in the Controlled Group.

Multimedia Appendix 2 The Real Variability of the Reference Sound Level Among Devices of the Same Model.

Multimedia Appendix 3 Estimation of the Standard Deviation of the Difference Between the Pure-Tone Audiometry and a Hearing Test Conducted on a Mobile Device Calibrated by Means of the Predefined Reference Sound Level.

## Abbreviations

CI: confidence interval
HL: hearing level
SD: standard deviation

## References

[1] Honeth L, Bexelius C, Eriksson M, Sandin S, Litton J, Rosenhall U, Nyrén O, Bagger-Sjöbäck D (2010). An internet-based hearing test for simple audiometry in nonclinical settings: preliminary validation and proof of principle. Otol Neurotol.

[2] Masalski M, Kręcicki T (2013). Self-test web-based pure-tone audiometry: validity evaluation and measurement error analysis. J Med Internet Res.

[3] Foulad A, Bui P, Djalilian H (2013). Automated audiometry using apple iOS-based application technology. Otolaryngol Head Neck Surg.

[4] Lee T, van HA, Kam Anna Chi Shan, Sung John Ka Keung, Wong Terence Ka Cheong (2012). Clinical evaluation of a computerized self-administered hearing test. Int J Audiol.

[5] Swanepoel DW, Myburgh HC, Howe DM, Mahomed F, Eikelboom RH (2014). Smartphone hearing screening with integrated quality control and data management. Int J Audiol.

[6] Szudek J, Ostevik A, Dziegielewski P, Robinson-Anagor J, Gomaa N, Hodgetts B, Ho A (2012). Can Uhear me now? Validation of an iPod-based hearing loss screening test. J Otolaryngol Head Neck Surg.

[7] Khoza-Shangase K, Kassner L (2013). Automated screening audiometry in the digital age: exploring uhear™ and its use in a resource-stricken developing country. Int J Technol Assess Health Care.

[8] Handzel O, Ben-Ari O, Damian D, Priel MM, Cohen J, Himmelfarb M (2013). Smartphone-based hearing test as an aid in the initial evaluation of unilateral sudden sensorineural hearing loss. Audiol Neurootol.

[9] Abu-Ghanem S, Handzel O, Ness L, Ben-Artzi-Blima M, Fait-Ghelbendorf K, Himmelfarb M (2016). Smartphone-based audiometric test for screening hearing loss in the elderly. Eur Arch Otorhinolaryngol.

[10] Folkeard P, Van Tasell Dianne J (2013). Reliability and accuracy of a method of adjustment for self-measurement of auditory thresholds. Otol Neurotol.

[11] McPherson B, Law M M S, Wong M S M (2010). Hearing screening for school children: comparison of low-cost, computer-based and conventional audiometry. Child Care Health Dev.

[12] Margolis RH, Morgan DE (2008). Automated pure-tone audiometry: an analysis of capacity, need, and benefit. Am J Audiol.

[13] Penteado SP, Bento RF, Battistella LR, Silva SM, Sooful P (2014). Use of the satisfaction with amplification in daily life questionnaire to assess patient satisfaction following remote hearing aid adjustments (telefitting). JMIR Med Inform.

[14] Wu C, Wu S, Chen P, Lin Y (2014). An innovative smartphone-based otorhinoendoscope and its application in mobile health and teleotolaryngology. J Med Internet Res.

[15] Swanepoel DW, Clark JL, Koekemoer D, Hall JW, Krumm M, Ferrari DV, McPherson B, Olusanya BO, Mars M, Russo I, Barajas JJ (2010). Telehealth in audiology: the need and potential to reach underserved communities. Int J Audiol.

[16] Fagan JJ, Jacobs M (2009). Survey of ENT services in Africa: need for a comprehensive intervention. Glob Health Action.

[17] Swanepoel DW, Mngemane S, Molemong S, Mkwanazi H, Tutshini S (2010). Hearing assessment-reliability, accuracy, and efficiency of automated audiometry. Telemed J E Health.

[18] Hildreth AJ, Lindsey L, Ho Allan Thiam Poh (2009). Computer-assisted audiometry versus manual audiometry. Otol Neurotol.

[19] Margolis RH, Glasberg BR, Creeke S, Moore Brian C J (2010). AMTAS: automated method for testing auditory sensitivity: validation studies. Int J Audiol.

[20] Wong TW, Yu TS, Chen WQ, Chiu YL, Wong CN, Wong A H S (2003). Agreement between hearing thresholds measured in non-soundproof work environments and a soundproof booth. Occup Environ Med.

[21] Kimball SH (2008). Inquiry into online hearing test raises doubts about its validity. The Hearing Journal.

[22] Choi JM, Lee HB, Park CS, Oh SH, Park KS (2007). PC-based tele-audiometry. Telemed J E Health.

[23] Senses Examination Platform.

[24] Masalski M, Grysiński T, Kręcicki T (2014). Biological calibration for web-based hearing tests: evaluation of the methods. J Med Internet Res.

[25] (2015). Gartner Newsroom.

[26] Masalski M (2014). Hearing Test App - Android Apps on Google Play.

[27] (2011). British Society of Audiology.

[28] Engdahl B, Tambs K, Borchgrevink HM, Hoffman HJ (2005). Screened and unscreened hearing threshold levels for the adult population: results from the Nord-Trøndelag Hearing Loss Study. Int J Audiol.

[29] Rousseeuw P, Leroy A (1988). A robust scale estimator based on the shortest half. Statistica Neerland.

[30] Bickel DR (2002). Robust estimators of the mode and skewness of continuous data. Computational Statistics & Data Analysis.

[31] Bickel DR (2003). Robust and efficient estimation of the mode of continuous data: the mode as a viable measure of central tendency. Journal of Statistical Computation and Simulation.

[32] Efron B, Tibshirani R (1993). An introduction to the bootstrap.

