# Supplementary figures and images for: Hearing Tests on Mobile Devices: Evaluation of the Reference Sound Level by Means of Biological Calibration
Source: J Med Internet Res. 2016 May 30;18(5):e130. doi: 10.2196/jmir.4987 (PMC4906240; doi:10.2196/jmir.4987)

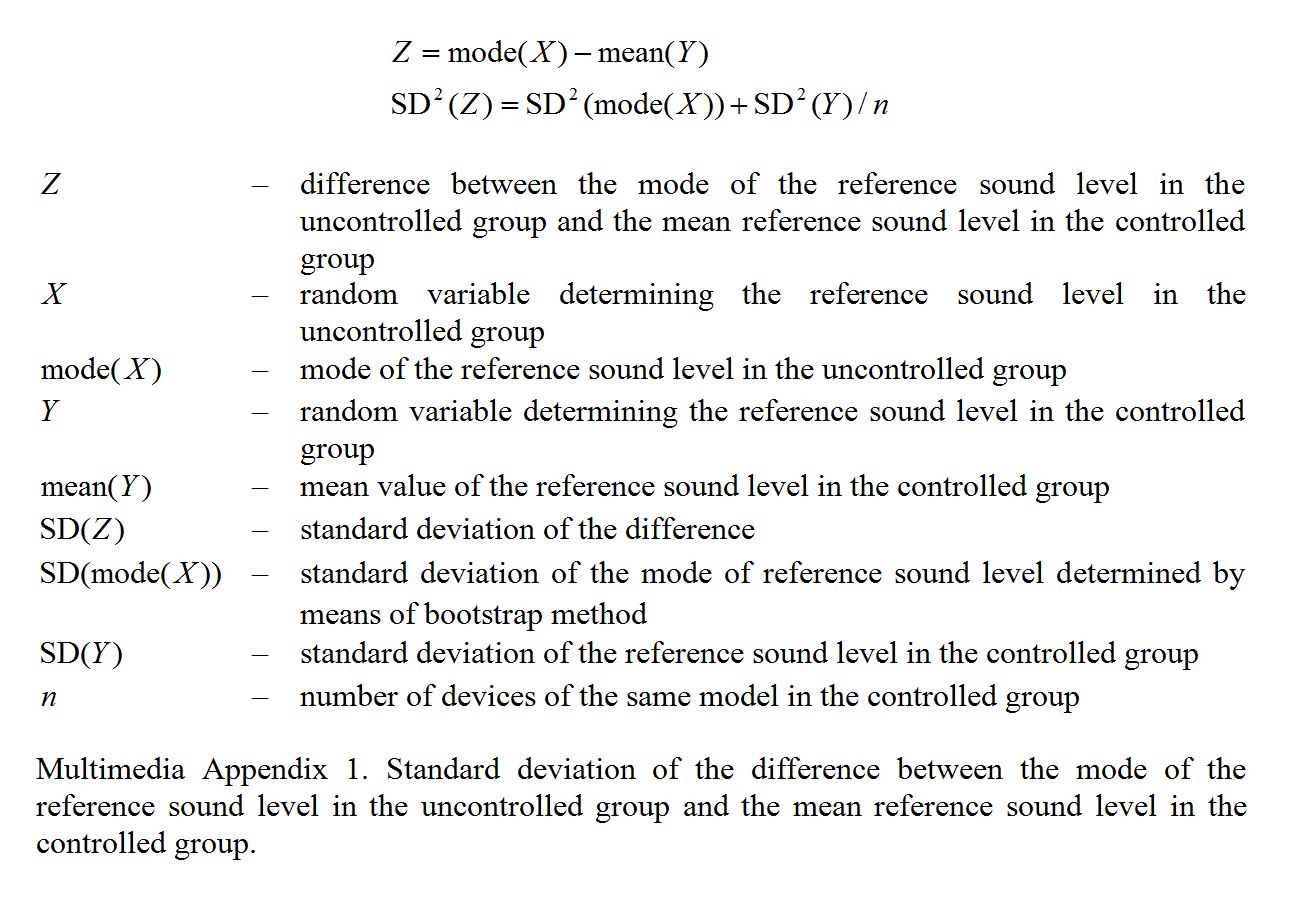

Supplement: Multimedia Appendix 1 [file jmir_v18i5e130_app1.png]

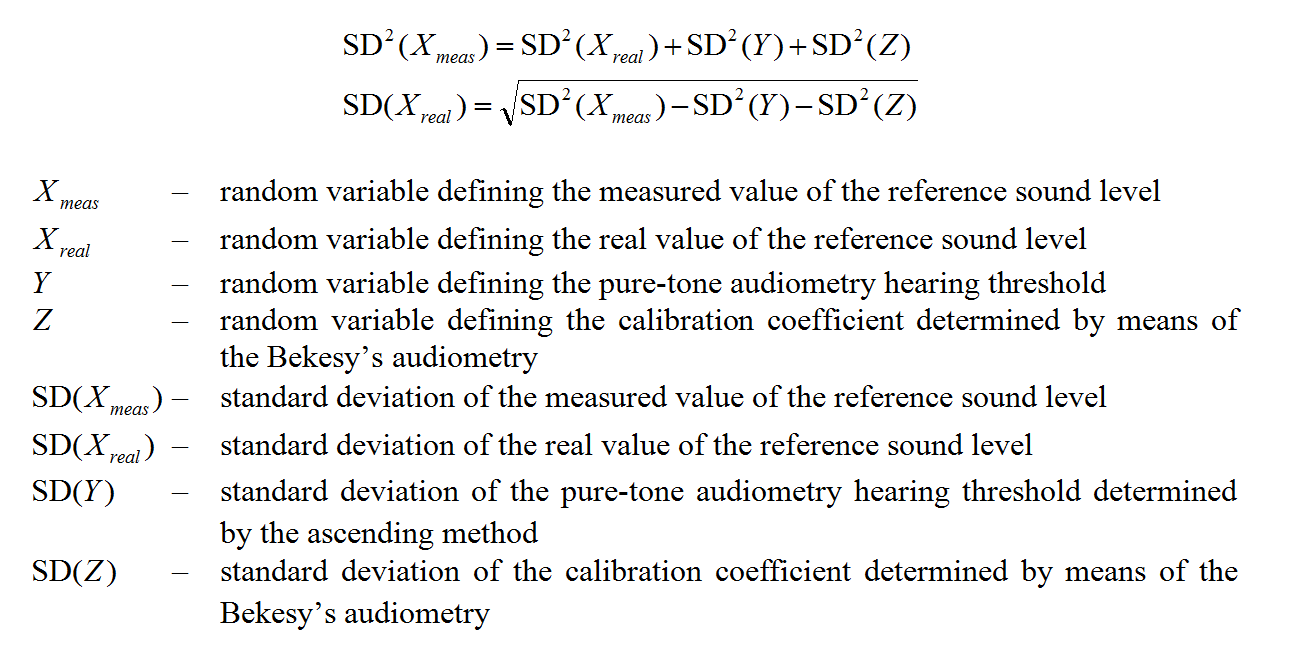

Supplement: Multimedia Appendix 2 [file jmir_v18i5e130_app2.png]

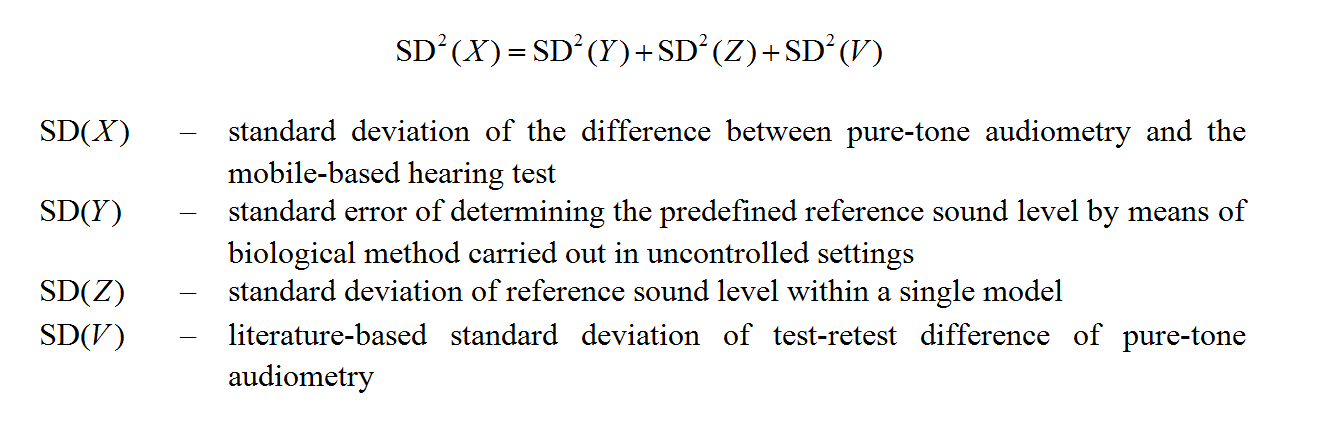

Supplement: Multimedia Appendix 3 [file jmir_v18i5e130_app3.png]
